# c-FLIP-Short Reduces Type I Interferon Production and Increases Viremia with Coxsackievirus B3

**DOI:** 10.1371/journal.pone.0096156

**Published:** 2014-05-09

**Authors:** Iwona A. Buskiewicz, Andreas Koenig, Brian Roberts, Jennifer Russell, Cuixia Shi, Sun-Hwa Lee, Jae U. Jung, Sally A. Huber, Ralph C. Budd

**Affiliations:** 1 Department of Pathology, Vermont Center for Immunology and Infectious Diseases, University of Vermont, Burlington, Vermont, United States of America; 2 Department of Medicine, Vermont Center for Immunology and Infectious Diseases, University of Vermont, Burlington, Vermont, United States of America; 3 Department of Molecular Microbiology and Immunology, University of Southern California, Los Angeles, California, United States of America.; University of British Columbia, Canada

## Abstract

Cellular FLIP (c-FLIP) is an enzymatically inactive paralogue of caspase-8 and as such can block death receptor-induced apoptosis. However, independent of death receptors, c-FLIP-Long (c-FLIP_L_) can heterodimerize with and activate caspase-8. This is critical for promoting the growth and survival of T lymphocytes as well as the regulation of the RIG-I helicase pathway for type I interferon production in response to viral infections. Truncated forms of FLIP also exist in mammalian cells (c-FLIP_S_) and certain viruses (v-FLIP), which lack the C-terminal domain that activates caspase-8. Thus, the ratio of c-FLIP_L_ to these short forms of FLIP may greatly influence the outcome of an immune response. We examined this model in mice transgenically expressing c-FLIP_S_ in T cells during infection with Coxsackievirus B3 (CVB3). In contrast to our earlier findings of reduced myocarditis and mortality with CVB3 infection of c-FLIP_L_-transgenic mice, c-FLIP_S_-transgenic mice were highly sensitive to CVB3 infection as manifested by increased cardiac virus titers, myocarditis score, and mortality compared to wild-type C57BL/6 mice. This observation was paralleled by a reduction in serum levels of IL-10 and IFN-α in CVB3-infected c-FLIP_S_ mice. *In vitro* infection of c-FLIP_S_ T cells with CVB3 confirmed these results. Furthermore, molecular studies revealed that following infection of cells with CVB3, c-FLIP_L_ associates with mitochondrial antiviral signaling protein (MAVS), increases caspase-8 activity and type I IFN production, and reduces viral replication, whereas c-FLIP_S_ promotes the opposite phenotype.

## Introduction

Coxsackievirus B3 (CVB3) is a single stranded, positive sense RNA virus that is one of the major etiological viral agents of human myocarditis and dilated cardiomyopathy [1]–[3]. The virus also rapidly infects the myocardium of mice, reaching peak viral titers within 3–4 days and then declining in the heart until eliminated, usually within 10–14 days [4]. Viral elimination depends upon several distinct host defense mechanisms including type I interferons (IFN-α and IFN-β) [5]–[8], T cell response to CVB3 [8], virus neutralizing antibody [9], and activated macrophages [10]. Several reports show that blocking type I IFN, either by injection of anti-interferon antibodies or use of IFN receptor α/β-deficient mice, results in greater viral burden and mortality [5], [8], [11], whereas administration of exogenous type I IFN ameliorates the disease [11], [12].

Although early inflammatory responses are important for resolution of virus infection, there is accumulating evidence to indicate that the cellular inflammatory infiltrate following viral infection is directly associated with disease severity in experimental models of viral myocarditis [13], [14]. High numbers of lymphocytes persisting in the myocardium can lead to exacerbation of disease. Thus, a delicate balance between the beneficial and detrimental effects of the immune response must be established to promote efficient protection. The type of immune cells involved in myocardial inflammation may ultimately lead to either the resolution or progression of disease. It was shown that IFN-β immunotherapy significantly reduces the principal CD8^+^ T cells that are found in the cardiac infiltrate during the chronic phase of autoimmune myocarditis following virus infection [15]. Therefore, better knowledge of the regulation of type I IFN production and its effects on myocardial infiltrates will assist in the development of therapeutic strategies to improve the prognosis of chronic inflammatory heart disease.

The recognition of viruses by the innate immune system depends largely on the ability to discriminate viral nucleic acids from host RNA or DNA. The major pattern recognition receptors for virus-derived RNA, originating from either genomic RNA or replication intermediates, are the retinoic acid-inducible gene I (RIG-I) and melanoma differentiation associated gene 5 (MDA5) helicases, which interact with a common adaptor, mitochondrial antiviral signaling molecule (MAVS, also known as VISA/IPS-1/Cardif) to activate NF-κB and IRF3 [16]–[18]. MAVS is localized to the mitochondrial membrane and to peroxisomes via a C-terminal transmembrane domain, which is essential for innate immune signaling. MDA5 and MAVS have been shown to be critical for initiation of the type I IFN response to coxsackievirus infection [8].

Viruses have evolved strategies to counter the activation of cellular defenses associated with microbial recognition in order to promote their replication and spread. Virally encoded proteases have been shown to directly target components of the innate immune system, and MAVS is known to be cleaved by proteases of hepatitis C, A and GB viruses, as well as by proteases of rhinovirus [19]–[22]. Coxsackievirus also harbors a 3C^pro^ cysteine protease that cleaves MAVS and ablates its signaling [23]. The 3C^pro^ cleavage site within MAVS (Q148) is located in the proline-rich region, which mediates its interaction with a number of signaling molecules, including TRAF-2, -3, and -6 [24], RIP1 [25] and FADD [16].

Accumulating evidence also points to a role for caspase-8 in innate immunity in addition to its well-established role in cell death following ligation of death receptors [26]. The first work linking caspase-8 to innate immunity showed that cells deficient in caspase-8 have reduced expression of inflammatory cytokines and NF-κB activation [27]. Other studies performed in keratinocytes revealed that deletion of caspase-8 resulted in an excessive activation of interferon regulatory factor 3 (IRF3) [28], which is consistent with subsequent studies describing a role of caspase-8-mediated RIP1 cleavage in restricting RIG-I signaling [29]. How caspase-8 activity is regulated in the RNA viral-sensing pathway is not entirely clear. Since caspase-8 is also critical for the activation and survival of T cells as well as other cell types [30]–[32], the regulation between death and growth processes is extremely important to T cell function and homeostasis.

A critical regulator of caspase-8 activation is the caspase-8 paralogue, c-FLIP [26]. Originally identified in certain DNA viruses (v-FLIP) [33], the cellular homolog (c-FLIP) was described a year later [34]. c-FLIP is expressed in three forms, full-length c-FLIP-Long (c-FLIP_L_), and two alternatively spliced forms that are upregulated following T cell activation, c-FLIP-Short (c-FLIP_S_) and c-FLIP-Reduced (c-FLIP_R_) [35], [36]. Since all three forms of c-FLIP, as well as v-FLIP, inhibit death receptor-induced activation of caspase-8 and cell death [26], it has been less clear what is the distinction, if any, among these various forms of c-FLIP. A clue to the explanation came when it was determined that c-FLIP_L_ can heterodimerize with caspase-8, independently of death receptor ligation, via their mutual Death Effector Domains (DED) [37]. In this complex, c-FLIP_L_ contains within its enzymatically inert C-terminus an activation loop for caspase-8. However, this loop is absent in c-FLIP_S_, c-FLIP_R_, and v-FLIP, even though they can also heterodimerize with caspase-8 by their N-terminal DED [37], [38]. Thus, c-FLIP_L_ emerges as an activator of caspase-8 during the initiation of cell growth of T cells (and perhaps other cell types), whereas the later upregulation of c-FLIP_S_, or the presence of v-FLIPs, would be predicted to promote reduced activation of caspase-8 and perhaps serve to terminate T cell growth or function. This model is consistent with our observations that increased expression of c-FLIP_L_ in T cells resulted in their hyperproliferation [39], augmented production of certain cytokines [40], and ability to protect mice from CVB3 infection [41], whereas increased expression of c-FLIP_S_ resulted in reduced activation of caspase-8 and NF-κB, as well as reduced T cell survival following antigen activation [38].

We thus examined the role of c-FLIP_S_ during infection with CVB3 and observed that, in contrast to mice expressing c-FLIP_L_ in the T cell compartment, c-FLIP_S_-transgenic mice were more susceptible to CVB3 infection, particularly female mice that are usually resistant. *In vitro* mechanistic studies using CVB3 infection of mouse embryonic fibroblasts (MEF) further revealed that c-FLIP_L_ promoted caspase-8 activation and interferon production, whereas c-FLIP_S_ and v-FLIPs did the opposite.

## Materials and Methods

### Ethics statement

This study was carried out in strict accordance with the recommendations in the Guide for the Care and Use of Laboratory Animals of the National Institutes of Health. The protocol was reviewed and approved by the University of Vermont Institutional Animal Care and Use Committee (Animal Welfare Assurance # A3301-01).

### Mice

C57BL/6 mice were housed and bred in the AAALAC approved animal facility at the University of Vermont (UVM). All of the studies have been reviewed and approved by UVM' Institutional Animal Care and Use Committee (IACUC). The here presented work involved merely tissue harvest from euthanized mice, without survival surgery. Euthanasia was performed by CO_2_ intoxication followed by thoracotomy. This method is consistent with the recommendations of the Panel of Euthanasia of the Veterinary Medical Association. Female mice were typically used at 5 to 7 weeks of age. c-FLIP_S_ was expressed transgenically in the T cell compartment as previously described [38]. Briefly, FLAG-tagged human c-FLIP_S_ cDNA was inserted into a target vector containing the *lck* proximal promoter and a downstream human growth hormone locus. Transgenic mice were screened by PCR of ear DNA for the human growth hormone sequence with the c-FLIP_S_ construct using the following primers:

5′ Primer: (5′-TAGGAAGAAGCCTATATCCCAAAGG -3′)

3′ Primer: (5′-ACAGTCTCTCAAAGTCAGTGGGG-3′).

### Virus strain, titers and infection of mice

The H3 variant of CVB3 was made from an infectious cDNA clone as described previously [42]. Mice were injected intraperitoneally (i.p.) with 10^2^ plaque forming units (PFU) virus in 0.5 ml PBS. Animals were killed when moribund or 7 days after infection. Hearts were aseptically removed from the animals, weighed, homogenized in RPMI 1640 medium containing 5% fetal bovine serum (FBS), L-glutamine, streptomycin and penicillin. Cellular debris was removed by centrifugation at 300×g for 10 min. Supernatants were diluted serially using 10-fold dilutions and titered on HeLa cell monolayers by the plaque forming assay [43]. To account that the mice were indeed infected we always checked the presence of the virus in pancreas from all mice as this organ is the most sensitive to CVB3 infection. If mice were successfully infected, acinar cell degranulation will be evident at day 7 post infection. Only mice with total acinar cell degradation were evaluated as proof that these animals actually were adequately infected with virus even if virus clearance occurred by day 7 in the heart. Total of 10 mice of each group: female, male, wild type, transgenic group, per experiment was used. Each experiment was repeated at least 3 times.

### Histology

Heart tissue was fixed in 10% buffered formalin for 48 hours, paraffin embedded, sectioned and stained by hematoxylin and eosin. Image analysis of cardiac inflammation was done as described previously [42].

### Isolation of cardiac myocytes

Isolation of neonatal mouse myocytes has been described previously [44]. Briefly, hearts were obtained from mice within 72 hours of birth, minced finely and subjected to sequential digestion with 0.25% pancreatin and 0.4% collagenase. The single cell suspension was washed and depleted of endothelial cells and fibroblasts by two sequential one hour adsorptions to plastic. The non-adherent population was retrieved and plated into 1-mm tissue culture wells at 10^3^ cells/well. After 48 hours at 37°C in a humidified 5% CO_2_ incubator, the wells were used for cytotoxicity assays.

### Isolation of lymphocytes and T cell purification

Spleens were removed and pressed through fine mesh screens. Lymphoid cells were isolated by centrifugation of cell suspensions on Histopaque. Spleen and lymph node cells were isolated and disrupted through nylon mesh in RPMI 1640 with 25 mM HEPES containing 5% (v/v) BCS. Erythrocyte lysis of splenocytes was performed using Gey' solution. T cells were purified using negative selection by incubating splenocytes with anti-MHC II (M5/114/15/2), anti-CD11b (M1/70), anti-NK1.1 (PK136), and anti-B220 (RA3-6B2) on ice for 30 min. Cells were washed three times and rocked with goat anti-mouse and goat anti-rat conjugated magnetic beads at a 10∶1 ratio of beads to cell at 4°C for 45 min. Magnetic depletion was used to remove bead-bound cells. Cells were washed and resuspended in culture medium RPMI 1640, 2.5 mg/ml glucose, 10 mg/ml folate, 110 µg/ml pyruvate, 5×10^−5^ M 2-mercaptoethanol, 292 µg/ml glutamine, 100 U/ml penicillin-streptomycin, and 5% FCS).

### T cell culture and *in vitro* infection of T lymphocytes

C57BL/6 and c-FLIP_S_-Tg T cells were activated in culture medium by plate-bound anti-CD3 (10 µg/ml, clone 145-2C11), anti-CD28 ascites (1∶500) and recombinant human IL-2 (50 U/ml) for 2 days. Cells were then removed from anti-CD3 and supplied with fresh medium plus IL-2. 10^4^ T cells were plated in 96 well round-bottom tissue culture plates and infected with 10^5^ PFU CVB3 for 30 min at 37°C in a total volume of 50 µl. The cells were washed twice with medium containing 10 µg/ml monoclonal anti-CVB3 (clone 8A6) and twice with medium alone, then cultured for up to 3 days in RPMI 1640 medium containing 10% FBS, L-glutamine and antibiotics. The cells were centrifuged at 325×g and the supernatants were removed for evaluation of IFN-α by ELISA. Remaining cells were resuspended in 100 µl fresh medium, followed by three cycles of freeze-thawing, and titered using serial 10-fold dilutions by the plaque forming assay on HeLa cell monolayers to determine virus titers.

### Cell-mediated cytotoxicity (CMC) assay

The CMC assay has been described in detail previously [44]. Targets were labeled with 100 μCi ^51^Cr (Na_2_^51^CrO_4_; ICN, Irvine, CA) for 2 hours at 37°C, washed 4 times and cultured for 12 hours at 37°C with 10^4^ lymphocytes (effector∶target ratio of 10∶1). Supernatants were removed and counted in a Packard Gamma Counter. Pellets were lysed using 6N HCl and the pellets were removed and counted. To determine maximal releasable ^51^Cr, some wells were directly treated with HCl to lyse all targets. Percent ^51^Cr release was calculated as: CPM in supernatant/(CPM in supernatant + CPM in pellet) ×100 for each well. Percent specific lysis represents: (% ^51^Cr release in experimental wells - % ^51^Cr release in medium control wells)/(% ^51^Cr in HCl lysed maximum release wells - % ^51^Cr release in medium control wells) ×100.

### Anti-CD8 treatment of lymphocytes

Spleen lymphocytes were incubated with 1 µg/ml of monoclonal anti-CD8 antibody (clone 53-6.7) and 1∶10 dilution of rabbit complement for 30 min at 37°C. The cells were centrifuged on Histopaque to remove dead cells. CD8 cell depletion was >95% as determined by flow cytometry.

### Flow cytometry and intracellular cytokine staining

Spleen lymphocytes were incubated with 1∶100 dilutions of FcBlock, PE-Cy5-anti-CD4 (clone GK1.5), FITC-anti-CD8 (clone 53–6.7) and PE-anti-CD69 (clone H1-2F3) for 30 min at 4°C, washed and fixed in 2% paraformaldehyde. All antibodies were purchased from BD Biosciences/Pharmingen. Intracellular cytokine staining has been published previously [45]. 10^5^ spleen cells were cultured for 4 hours in RPMI 1640 medium containing 10% fetal bovine serum, antibiotics, 10 µg/ml of Brefeldin A (BFA), 50 ng/ml phorbol myristate acetate, and 500 ng/ml ionomycin. The cells were washed in PBS-1% bovine serum albumin (BSA) containing BFA, incubated on ice for 30 min in PBS-BSA-BFA containing a 1∶100 dilution of Fc Block, and PerCP-Cy5.5 anti-CD4 (clone GK1.5) or PerCP-Cy5.5 rat IgG2b (clone A95-1). For detection of intracellular IFN-α in CD4^+^ and CD8^+^ T cells or plasmacytoid dendritic cells, the surface staining cocktail contained 1∶100 dilutions of Fc Block and APC anti-CD11c (clone HL3), PE anti-B220 (clone RA3-GB2), APC-Cy7 anti-CD8 (clone 53-6.7) and PerCP-Cy5.5 anti-CD4 (clone RM4-5). The cells were washed once with PBS-BSA-BFA, fixed in 2% paraformaldehyde for 10 min, then resuspended in PBS-BSA containing 0.5% saponin, Fc Block and 1∶100 dilutions of the following antibodies or isotype control immunoglobulins: PE anti-IFN-γ (cloneXMG1.2) or PE-rat IgG1 (clone R3-34); FITC anti-IFN-α (clone RMMA-1, PBL Interferon Source), or FITC-rat IgG1 (clone R3-34), FITC anti-IL-10 (clone JES5-16E3) or FITC-rat IgG2b (clone A95-1); FITC-anti-IL-4 (clone 11B11) or FITC-rat IgG1 (clone R3-34) and incubated for 30 min on ice. All antibodies were from BD Biosciences/Pharmingen unless indicated otherwise. FoxP3 labeling was done using the eBioscience kit according to manufacturer' directions. Cells were labeled with Alexa647 anti-CD4 and PerCP-Cy5.5 anti-CD25 (clone PC61) in PBS-1%BSA containing FcBlock, washed, fixed and permeabilized, then incubated with PE-anti-FoxP3 and FcBlock overnight at 4°C. The cells were washed once in PBS-BSA-saponin and once in PBS-BSA, the resuspended in 2% paraformaldehyde. Cells were analyzed using a BD LSR II flow cytometer with a single excitation wavelength (488 nm) and band filters for PerCP-Cy5.5 (695/40 nm), FITC (525 nm), and PE (575 nm). The excitation wavelength for Alexa 647 is 643 nm and a band filter of 660/20 nm was used for detection. The cell population was classified for cell size (forward scatter) and complexity (side scatter). At least 10,000 cells were evaluated. Positive staining was determined relative to isotype controls. To determine the number of individual cell populations, the total number of viable cells was determined by trypan blue exclusion. Following flow cytometry, the % of a subpopulation staining with a specific antibody was multiplied by the total number of cells.

### Isolation of plasma and analysis of cytokines and chemokines by Bio-Plex

Mice were anesthetized using pentobarbital, the thoracic cavity opened and blood was removed by intracardiac puncture using a 1 cc syringe containing 0.05 ml saturated EDTA. Plasma was obtained by centrifugation of the blood at 325×g for 20 min. Plasma cytokine levels were detected using Bio-Rad Mouse 23-Plex Panel according to the manufacturer's directions. Samples were collected and analyzed by a Bio-Plex 200 System instrument.

### ELISA for IFN-α and -β

IFN concentration in plasma or tissue culture supernatants was determined using the Mouse Interferon-Alpha or Beta ELISA kit (PBL Biomedical Laboratories) according to manufacturer' directions.

### Mouse embryonic fibroblasts culture

MEFs with various c-FLIP, v-FLIP or c-FLIP^−/−^ were a kind gift of Prof. Jung and were described in detail in Lee et al., 2009 [46]. MEF with knocked out caspase-8 and reconstituted with non-cleavable version of caspase-8 were a kind gift of Prof. Frisch and described in Helfer et al., 2006 [47]. The MEF caspase-8 knockout and reconstituted MEF overexpressing c-FLIP_L_ and c-FLIP_S_ were established as stably transfected cell lines with vectors described in Lee et al., 2009 [46]. The MEF MAVS knockout cells were a kind gift of Prof. Chen and described in Seth et al., 2005 [18].

### Biotin-VAD-fmk caspase-8 precipitation assay, FLAG precipitation and western blotting

MEFs (3×10^6^) were cultivated in 10 cm dishes and infected with CVB3 (the titer of the CVB3 stock was 10^8^ plaque-forming-units (pfu/ml), MEFs were infected with 10^−5^ dilution). 24 h post infection cells were incubated with 50 µM bVAD–fmk (SMBiochemicals), or DMSO control for 5 h at 37°C. The cells were then scraped, quickly washed in PBS and lysed. We have tested several lysis buffers to ensure that whole cell lysates contain caspase-8 from the mitochondrial fraction, and that the treatment does not cause damage to mitochondria and proteolysis of MAVS. After lysis, a total of 600 ug of protein per cell line was mixed with 20 µl of magnetic streptavidin-beads (Milipore) and incubated at 4°C for 2 h. The beads were then washed 5 times with lysis buffer and active caspase-8 was then eluted by boiling the beads for 5 min at 100°C and resolved by SDS–PAGE. Caspase-8 was detected by immunoblotting. Total cellular caspase-8 activity in each cell line was quantitated as well using DEVD-rhodamine (Casp8-Glo - Promega) according to the manufacturer' protocol. The endogenously biotinylated protein acetyl-CoA carboxylase functioned as a control for both pull-down efficiency and for loading (data not shown). The FLAG precipitation was performed with ANTI-FLAG M2 Magnetic Beads on total of 300µg of protein per cell line. The fraction of active caspase-8 or cleaved MAVS was quantified with Quantity One 4.5.0 (BioRad).

### Quantitative real-time PCR to detect positive- and negative-sense CVB3 RNAs and type I IFN

pDC, CD4 and CD8 positive cells from uninfected control mice and infected animals were isolated from spleens and sorted by flow cytometry. RNA was isolated from the same number of cells per sample using the RNeasy kit (Qiagen) according to the manufacturer' instructions. A total of 0.5µg of total RNA per sample was reverse transcribed using SuperScript III reverse transcriptase (Invitrogen) and oligo(dT)18 or primer according to the manufacturer' protocol and as described previously. Because of low CVB3 titer of 10^2^ PFU used for infection, to increase the sensitivity of CVB3 RNA detection, we separately enriched for ssRNAs and dsRNAs by LiCl differential precipitation from mock- and CVB3-infected cells as described previously [48]. The RT reactions were carried out as described previously [49], [50]. Subsequently, we carried out TaqMan quantitative real-time PCR using previously published CVB3-specific primers (forward primer: 5′CACACTCCGATCAACAGTCA-3′; reverse primer: 5′GAACGCTTTCTCCTTCAACC-3′) and a 6-carboxyfluorescein (FAM)/6-carboxytetramethylrhodamine (TAMRA)-labeled probe (5′-CGTGGCACACCAGCCATGTTT-3′) as previously described [49], [50]. PCR amplification was done using Platinum quantitative PCR SuperMix-UDG ready-to-use cocktail (Invitrogen) and quantitative analysis of viral RNA was carried out using a Bio Rad CFX96 Real-Time PCR Detection System as described previously [49], [50]. The ratio of genome copy number to the cycle threshold value was obtained from a standard curve, which was generated from a known quantity of *in vitro*-transcribed CVB3 genomic RNA, serially diluted, which was subjected the reverse transcriptase and quantitative PCR. The values in the Figures are expressed as the average numbers of CVB genome copies per µg RNA. All samples, including enriched and total RNA were evaluated in 5 parallel amplification reactions from 3 infections.

Specific primers for mouse IFN-α subtypes, namely, IFN-α1, IFN-α4 and IFN-β were used as described previously [51]. The primers for CD55 were obtained from Qiagen. Data was analyzed using the 2^ΔΔCt^ method as previously described using β-actin as the calibrator gene [52]. Identification of CVB3 RNA by RT-PCR was performed as described previously [53], [54].

### Virus titer and infectious center assay (ICA)

The concentration of infectious particles released into the cell culture supernatant of MEF after 24 h was quantified with HeLa cells seeded one day prior to plaque assay at a density of 0.3×10^6^/ml in 6 cm culture dishes and incubated with 200µl of 10-fold serial dilutions from supernatants. After one hour, plates were washed with PBS and overlayed with 0.6% SeaPlaque agarose in growth medium. After 2 days cells were fixed for one hour with 10% formaldehyde, the agarose plaque was removed and the cell layer was stained with 0.5% crystal violet in 20% ethanol for 2 min. The plaques were quantified by visual counting. Furthermore, to determine the number of CD4^+^, CD8^+^ and plasmacytoid dendritic cells (pDC) cells that were productively infected *in vivo*, the isolated cells were washed from any adherent virus, serially diluted and 100 µl aliquots were added to HeLa cell monolayers. The monolayers, once settled, were overlaid with 0.6% agar, stained after 48 h with crystal violet and plaques were counted.

### Statistics

Differences between groups were determined by Wilcoxon Ranked Score and statistical significance was determined by one-way analysis of variance (ANOVA) (GraphPad Prism 6). p values 0.05, 0.01, and 0.001 were marked with *, ** and ***, respectively.

## Results

### c-FLIP_S_ mice manifest increased susceptibility to CVB3-induced myocarditis

**Figure 1:**
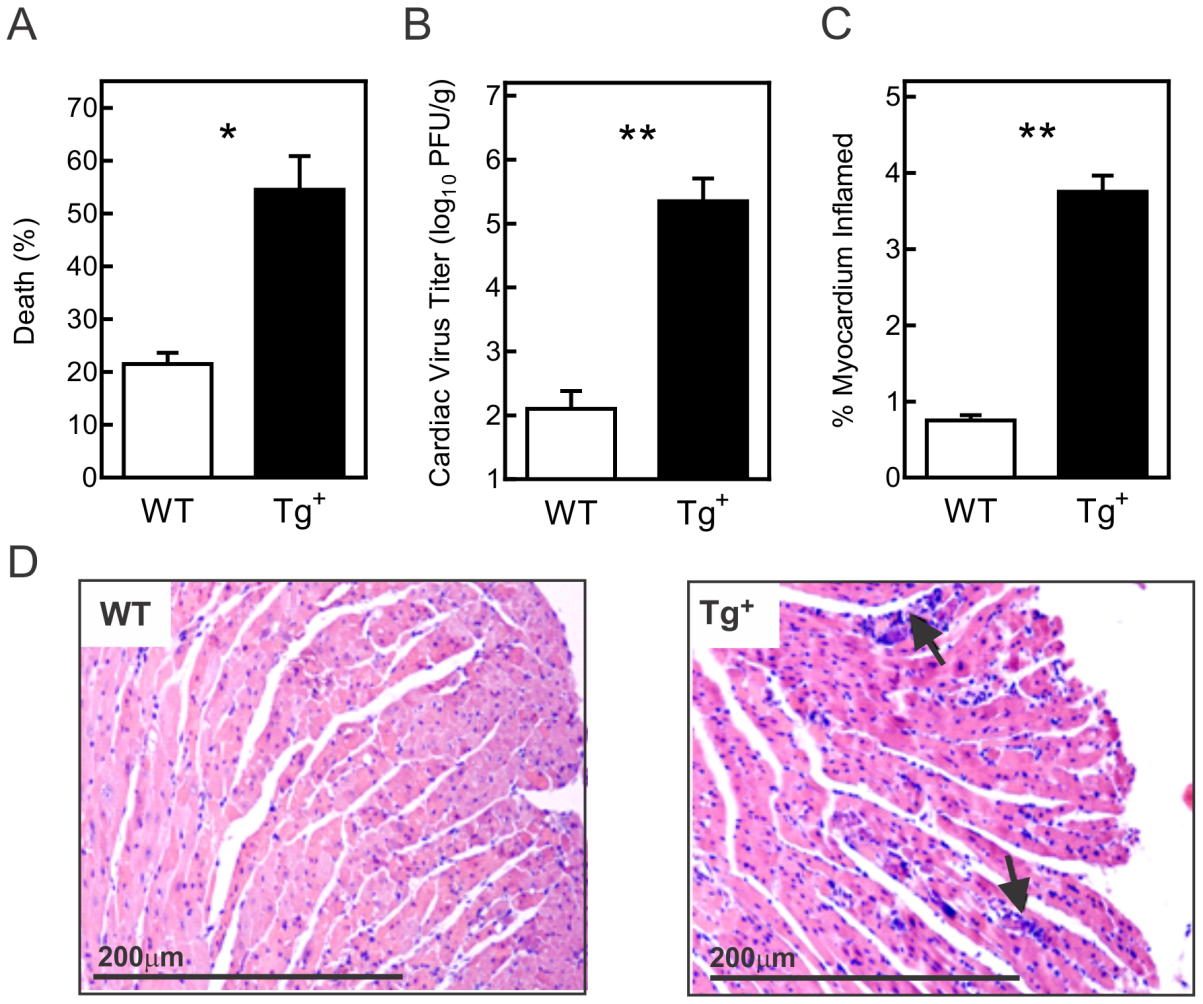
CVB3 infection of c-FLIP_S_-Tg female mice yields increased pathogenicity. Female c-FLIP_S_-transgenic (Tg^+^) and wild-type (WT) mice were infected with 10^2^ PFU CVB3 and surviving mice were euthanized 7 days after infection. (A) Cumulative mortality for the 7 day incubation period pooled from three different experiments. (B) Cardiac virus titers were determined by plaque forming assay. (C,D) Hearts were stained with hematoxylin and eosin and evaluated by image analysis for myocardial inflammation as described previously [42]. Representative histology is shown in (C) with arrows indicating inflammatory infiltrates, and cumulative score over all experiments shown in (D). Results are mean ± SEM of 6–8 mice/group. (*, **significantly different from c-FLIP_S_ mice at p<0.05 and p<0.01, respectively).

Male and female c-FLIP_S_-transgenic-positive and littermate control c-FLIP_S_-negative mice were infected with 10^2^ PFU CVB3, and surviving mice were euthanized 7 days later. Mortality was significantly increased in both male and female c-FLIP_S_ animals (8/20; 40%) compared to non-transgenic littermate control mice (4/20; 20%; p<0.05) (Fig. 1A). Consistent with the mortality rate, surviving c-FLIP_S_ mice had over 3 orders of magnitude higher viral titers in the heart and substantially greater cardiac inflammation compared to control animals (Fig. 1B, C). Representative histology for female mice is shown in Figure 1D.

CVB3 is known to cause acute myocarditis in several mouse strains, but is limited in wild-type C57BL/6 mice, especially in female mice, which are particularly resistant to viral infection and myocarditis. However, female c-FLIP_S_ mice become much more susceptible to CVB3 infection. To account that the mice were indeed infected we checked the presence of the virus in pancreas from all mice as this organ is the most sensitive to CVB3 infection. If mice were successfully infected, acinar cell degranulation will be evident at day 7 post infection. Only mice with total acinar cell degradation were evaluated as proof that these animals actually were adequately infected with virus. Since the female c-FLIP_S_ mice show strongest change in phenotype all the experiments presented are originating from female animals if otherwise stated.

**Figure 2:**
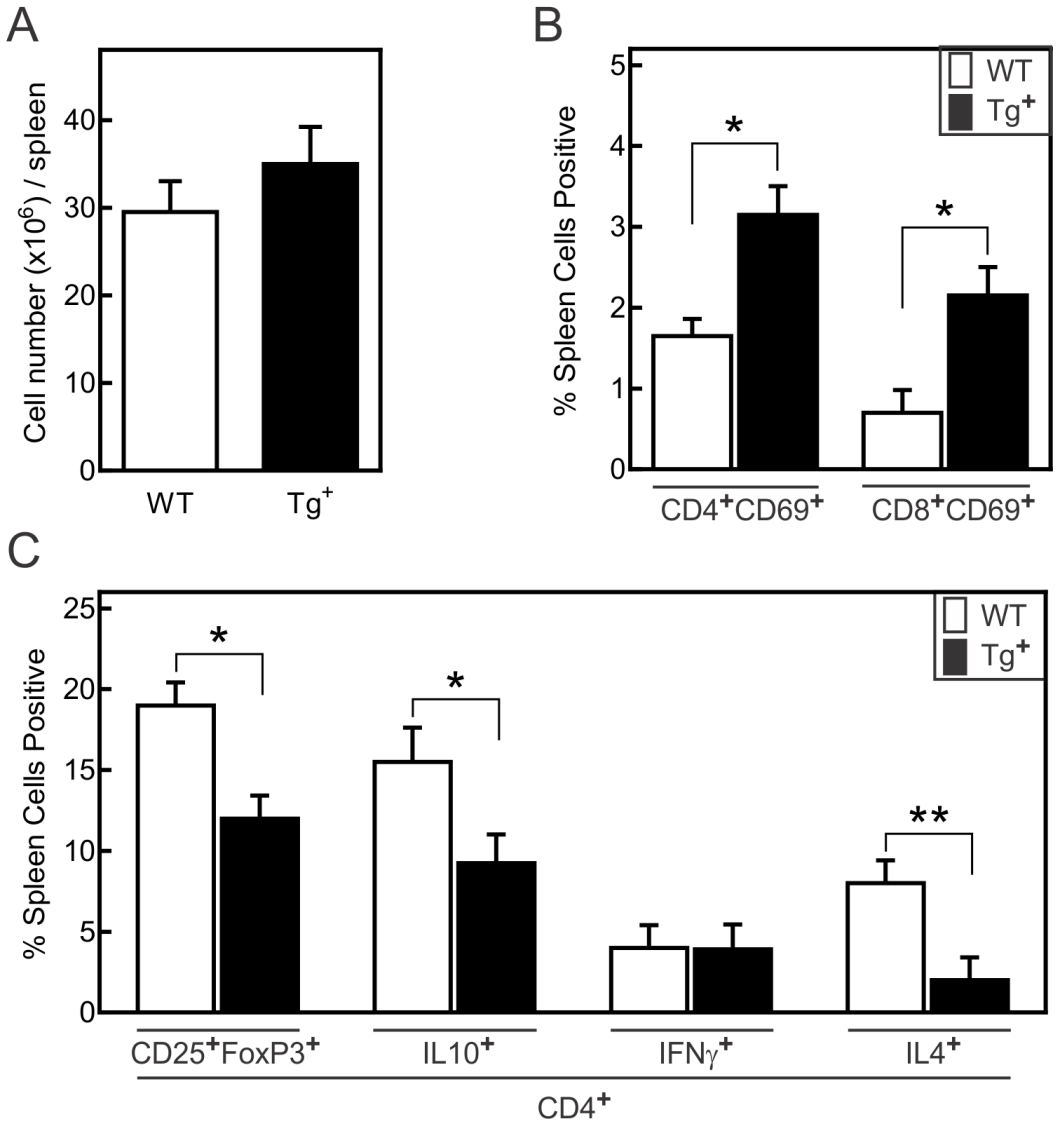
Activation state and cytokine production of T cells during CVB3 infection. Female c-FLIP_S_-transgenic (Tg^+^) and wild-type (WT) mice were infected with 10^2^ PFU CVB3 and surviving mice were euthanized 7 days after infection. (A) Total spleen cell numbers of day 7 surviving mice. (B) Spleen cells were isolated from individual mice, stained with antibodies to CD4, CD8, and the early activation marker, CD69, and evaluated by flow cytometry for the percent of cells doubly labeled for the indicated cell surface molecules. (C) Spleen cells were surface stained with antibodies to CD4 and CD25 and intracellularly labeled with antibodies to FoxP3, IL-10, IFN-γ or IL-4, then evaluated by flow cytometry for the percent of total spleen cells expressing the indicated molecules. Results are mean ± SEM of 6–8 mice/group. (*, **significantly different from c-FLIP_S_-Tg^−^ mice at p<0.05 and p<0.01, respectively).

The total number of spleen cells in infected mice showed no significant difference between the c-FLIP_S_ and control groups, although there was a tendency toward increased numbers in c-FLIP_S_ animals (Fig. 2A). However, FLIP_S_ mice manifested a greater proportion of activated CD69^+^ CD4^+^ and CD8^+^ T cells during CVB3 infection (Fig. 2B). c-FLIP_S_ mice also had a significantly reduced percentage of T regulatory (CD4^+^CD25^+^FoxP3^+^) cells as well as fewer CD4^+^IL-10^+^ and CD4^+^IL-4^+^ cells than control mice, as assessed by flow cytometry (Fig. 2C). The percentage of CD4^+^IFN-γ^+^ spleen cells was not different between the two groups. These results demonstrated that increased expression of c-FLIP_S_ specifically in T lymphocytes greatly enhanced myocarditis susceptibility in female mice, which may have resulted in part from a decreased proportion of immunosuppressive T cells.

### Cytokine/chemokine responses in c-FLIP_S_ mice and littermate control animals

**Figure 3:**
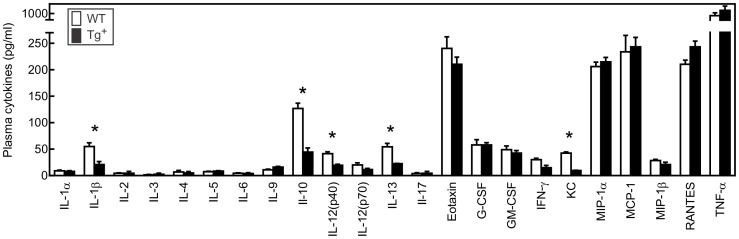
Cytokine and chemokine responses in c-FLIP_S_ female mice to CVB3 infection. Mice were infected with 10^2^ PFU CVB3 7 days earlier. Plasma was obtained from individual mice and evaluated for the indicated cytokines and chemokines by Bio-Plex. Results represent mean ± SEM of 5–11 mice/group. (*significantly different from c-FLIP_S_-Tg^+^ mice at p<0.05).

To further examine the effects of increased c-FLIP_S_ expression in T cells on the immune response to CVB3, we initially evaluated plasma levels of cytokines and chemokines during CVB3 infection. As shown in Figure 3, the dominant cytokine in the plasma was TNFα. There was no significant difference in TNFα concentrations between c-FLIP_S_ and control mice. Chemokines, including Eotaxin, RANTES, MCP-1 and MIP-1α, were also equivalently increased in both sets of mice during CVB3 infection. However, the chemokine, KC, was significantly decreased in c-FLIP_S_ mice as were the cytokines IL-1β, IL-10, IL-13 and IL-12p40 (Figure 3). These data indicate that circulating levels of several chemokines and cytokines did not greatly differ between the two groups of mice despite substantial differences in cardiac inflammation and cardiac virus titers.

**Figure 4:**
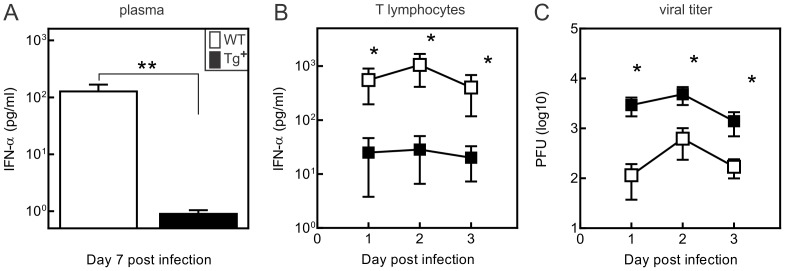
Reduced plasma IFN-α and increased viral burden in c-FLIP_S_ female mice infected with CVB3. (A) IFN-α concentration in plasma from CVB3-infected wild-type and c-FLIP_S_ female mice. Plasma was obtained from individual mice 7 days after infection and evaluated for IFN-α by ELISA. Results are mean ± SEM. (**significantly different from c-FLIP_S_ mice at p<0.05). (B) IFN-α production and (C) CVB3 viral titers in T cells *in vitro*. 10^4^ T lymphocytes from c-FLIP_S_ and wild-type mice were infected with 10^5^ PFU CVB3 for 30 min and extracellular virus was neutralized using monoclonal anti-CVB3 antibody. The infected cells were cultured for up to 3 days and intracellular viral titers were determined by plaque-forming assay, and IFN-α production determined by ELISA at the times indicated following infection. Results represent mean ± SEM of 4 replicate cultures. (*significantly different from wild type cells at p<0.05).

Given the known prominent role of type I IFN in controlling CVB3 infection [5], [8], [11], we further examined this parameter in c-FLIP_S_-infected mice. Plasma IFN-α levels in CVB3-infected mice showed no detectable levels in any c-FLIP_S_ mice, whereas IFN-α was readily detectable in all CVB3-infected non-transgenic littermate control mice (Fig. 4A). In order to determine whether c-FLIP_S_ might abrogate type IFN expression in T cells, splenic T cells were isolated from c-FLIP_S_ and control mice and incubated with 10 PFU/cell for up to 3 days. Supernatants were evaluated for IFN-α and found to be considerably lower in T cells from c-FLIP_S_ mice (Fig. 4B). An additional aliquot of T cells was homogenized and viral titers determined. Viral titers were significantly higher in cultures of T cells from c-FLIP_S_ mice compared to control T cells (Fig. 4C), in parallel with the reduced IFN-α production.

### Increased autoimmune CD8^+^ T cell response in c-FLIP_S_ mice

**Figure 5:**
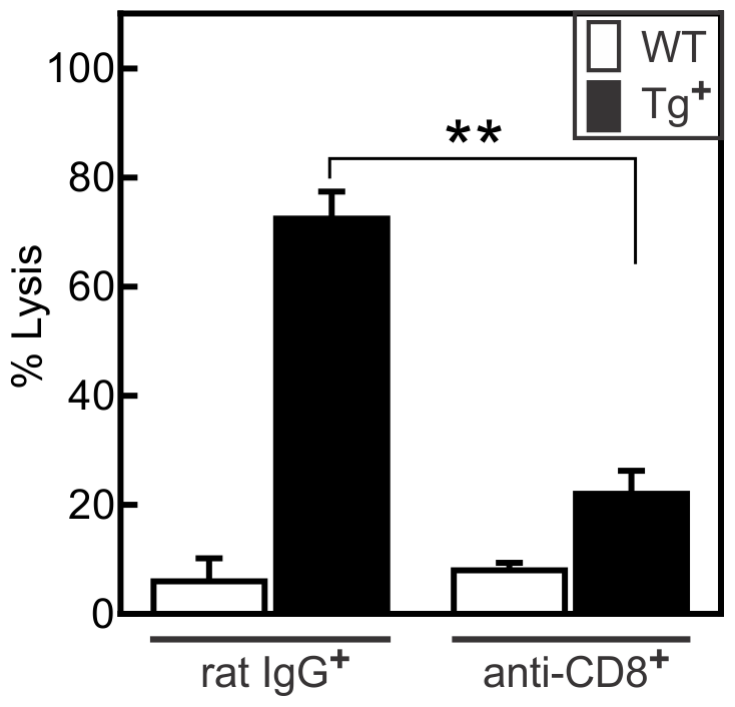
Increased cytolytic CD8^+^ T cell response in c-FLIP_S_ female mice infected with CVB3. Splenic lymphocytes were treated with either monoclonal anti-CD8 or rat IgG (1 µg/ml) and a 1∶10 dilution of rabbit complement for 30 min. Cells were then co-cultured with ^51^Cr-labeled cardiac myocytes at a 10∶1 effector∶target cell ratio for 12 h. Cytolysis was determined by the ^51^Cr release into the culture supernatant. Results represent mean ± SEM of 4 replicate cultures per group. (**significantly different from the c-FLIP_S_-Tg^−^ group at p<0.05).

CVB3 infection induces autoimmune CD8^+^ effector cells in male wild-type mice, which are the major cause of cardiac injury in this sex, but these autoimmune T cells are undetectable in infected female wild-type mice [55]–[60]. To determine if increased myocarditis susceptibility correlates to enhanced autoimmune CD8^+^ cell activation in c-FLIP_S_ mice, spleen cells were isolated from CVB3 infected c-FLIP_S_ and control female mice 7 days after infection and co-cultured with ^51^Cr-labeled syngeneic cardiac myocytes for 18 h. No cytolytic activity was found using spleen cells from control female mice, but lymphocytes from c-FLIP_S_ mice were highly cytolytic (Fig. 5). To confirm that the cytolytic activity was due to CD8^+^ cells, spleen cells were treated with monoclonal anti-CD8 antibody and complement prior to culture with the myocyte targets. Depletion of CD8^+^ cells nearly completely abrogated killing (Fig. 5).

### c-FLIP_S_ decreases type I IFN secretion and promotes CVB3 burden in mouse embryonic fibroblasts

**Figure 6:**
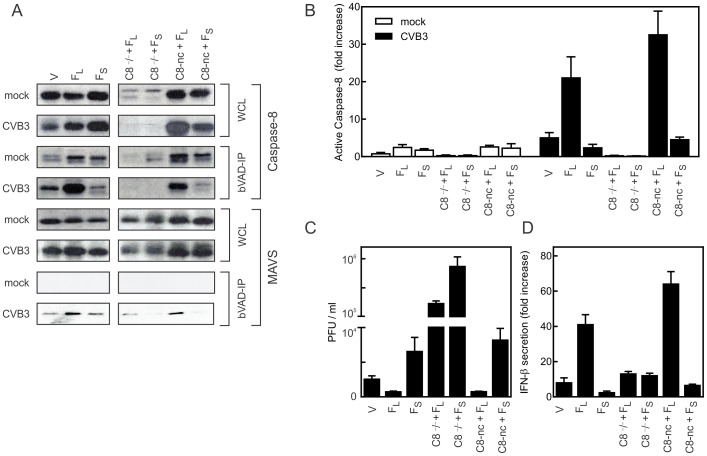
c-FLIP_L_ and c-FLIP_S_ oppositely modulate caspase-8 activity, IFN-β response, and viral replication, during CVB3 infection. (A) Immunoblot analysis of whole cell lysate (WCL) and biotin-VAD precipitate (bVAD-IP) for active caspase-8 in MEFs expressing vehicle plasmid (V), c-FLIP_L_ (F_L_), c-FLIP_S_ (F_S_), or caspase-8-deficient MEFs (C8−/−) reconstituted with non-cleavable caspase-8 (C8-nc) and/or FLIP variants. Cells were examined following mock infection (mock) and 24 h post infection with CVB3. Lysates and precipitates were examined for expression of caspase-8 and MAVS. (B) Caspase-8 activity from b-VAD precipitates was quantified by densitometry using Quantity One 4.5.0 software and plotted as fold change compared to uninfected vector control. (C, D) MEF supernatants were examined 24 h post-infection for release of CVB3 virus particles as plaque forming units (PFU) (C) and production of IFN-β (D).

To investigate the molecular mechanism responsible for the decreased type I IFN secretion during CVB3 infection in c-FLIP_S_ mice, we compared the interactions of c-FLIP_L_ and c-FLIP_S_ with caspase-8 and MAVS in mouse embryonic fibroblasts (MEFs). This included MEFs lacking caspase-8, or reconstituted with a non-cleavable (D387A) variant of caspase-8 (nc-C8) which lacks the initial self-processing site that separates the two subunits of the catalytic domain. The caspase-8 D387A processing mutant has previously been shown to rescue caspase-8-deficient T cell proliferation, verifying that caspase-8 self-processing is not required for its non-apoptotic function(s) [61]. To assess the caspase-8–independent function of c-FLIP as well as the role of caspase-8 cleavage in type I IFN induction, MEFs deficient in caspase-8 and reconstituted with non-cleavable caspase-8, were further stably transfected with c-FLIP_L_ or c-FLIP_S_. Since c-FLIP is known to heterodimerize with and regulate caspase-8 activation, we initially assessed the level of active caspase-8 by biotin-VAD precipitation using streptavidin-magnetic beads. The results shown in Figure 6A and B demonstrate that, upon CVB3 infection, the highest caspase-8 activity was observed in cells expressing c-FLIP_L_, which paralleled increased IFN-β secretion (Fig. 6D). These data are consistent with earlier observations that transgenic mice expressing c-FLIP_L_ in T cells respond better to viral infections and have higher plasma levels of IFN-β [41]. By contrast, c-FLIP_S_ decreased caspase-8 activity and IFN-β secretion during CVB3 infection. Caspase-8-deficient cells expressing either c-FLIP_L_ or c-FLIP_S_ manifested greatly decreased IFN-β secretion and high viral titers (Fig. 6C, D). These findings were consistent in three independent experiments, and confirmed the requirement of caspase-8 to induce IFN-β production during CVB3 infection, restoration with non-cleavable caspase-8 D387A restored caspase-8 activity, which was augmented by c-FLIP_L_ and reduced by c-FLIP_S_ (Fig. 6B).

We also tested whether FLIP isoforms associate with the MAVS complex by co-immunoprecipitation with the various FLAG-tagged FLIPs. We did not observe any association of MAVS with c-FLIP in uninfected cells (Fig. 6A bottom panels). However, following CVB3 infection MAVS was associated with the active caspase complex in biotin-VAD precipitates. This interaction was enhanced by c-FLIP_L_ and reduced by c-FLIP_S_ to the level of the vector control. Interestingly, the association of c-FLIP_L_ with MAVS was enhanced by the presence of caspase-8, as MAVS did not associate strongly with c-FLIP_L_ in caspase-8-deficient cells, whereas the association was increased in cells reconstituted with non-cleavable caspase-8. These findings suggested that FLIP influences the composition and function of the MAVS complex and secondarily the downstream activation of type I IFN.

### CVB3 infects CD4^+^, CD8^+^ and plasmacytoid dendritic cells

**Figure 7:**
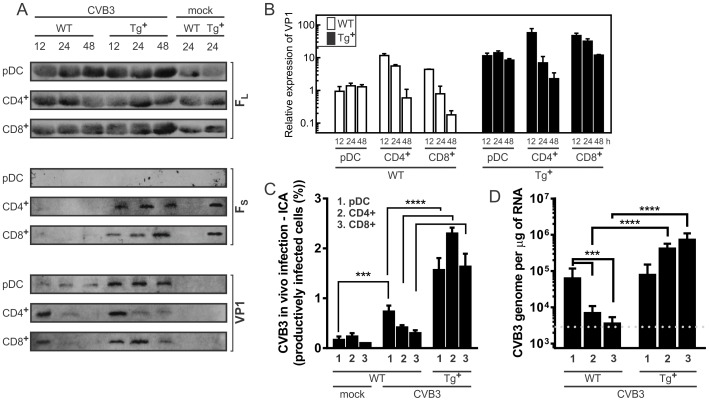
Increased CVB3 gene expression in lymphoid cell subsets of c-FLIP_S_ female mice. Immunoblot analysis of c-FLIP_L_, c-FLIP_S_, and CVB3 capsid protein VP1 expression in (A) plasmacytoid DC (pDC), CD4^+^, and CD8^+^ T lymphocytes purified from spleens of wild-type (WT) and c-FLIP_S_ (Tg^+^) mice. The cell subsets were purified from mock-infected or CVB3-infected mice at 0, 12, 24, and 48 h post-infection. (B) Relative expression of VP1 in different cell subsets was quantified from western blots by densitometry using Quantity One 4.5.0 software and plotted as fold change compared to VP1 detected in pDC isolated from wild-type mice 12 h after CVB3 infection. (C) Infectious Center Assay (ICA) for CVB3 infection of CD4^+^, CD8^+^ T cells and pDC. (D) Real-time PCR quantitation of viral genome copy number in CD4^+^, CD8+ T cells and pDC (Red dashed indicates line limit of detection). The data were obtained from wild-type and transgenic female animals (3 per group). Data were analyzed by the 2^ΔΔCt^ method using β-actin and / or ribosomal RNA as the calibrator.

We determined whether CVB3 infects CD4^+^, CD8^+^ and plasmacytoid dendritic cells (pDC). The different cell types were isolated from spleens of mice before and at 12, 24 and 48 h after infection with CVB3. Of note is that CD4^+^ and CD8^+^ T cells from c-FLIP_S_ mice expressed similar levels of the CVB3 receptor CD55 (data not shown). Before preparing cell lysates for western blot or RNA purification, the cells were treated with trypsin to remove any potentially adherent virus. Western blot analysis confirmed the transgenic expression of c-FLIP_S_ in T cells, which did not affect the expression of endogenous c-FLIP_L_ (Fig. 7A, B). Following CVB3 infection, VP1 viral protein was detected in all three types of cells. The pDC cells showed a steady presence of VP1 protein over the 48 h duration of infection. VP1 became detectable in CD4^+^ or CD8^+^ from wild-type mice as early as 12 h after infection. In the same cells isolated from c-FLIP_S_ mice the level of VP1 protein was at least one order of magnitude higher in pDCs and two orders of magnitude higher in CD4^+^ and CD8^+^ T cells. Although the presence of VP1 in CD4^+^ and CD8^+^ T cells from c-FLIP_S_ mice followed a similar rate of decline as observed for the wild-type T cells, the viral protein was still detectable in c-FLIP_S_ CD4^+^ cells at 24 h and in CD8^+^ cells lasted up to 48 h (Fig. 7A, B). The absence of detectable VP1 expression with time could be attributed either to the loss of viral material from the cells, or the viral genome might persist, but not expressed.

To determine whether or not CVB3 productively infects CD4^+^, CD8^+^ T cells, or pDC, we used the previously described by other groups for CVB3 infectious center assays (ICA) [49]. In brief, cells from spleens of infected mice, and from uninfected controls, were sorted and treated with trypsin to remove any possible adherent virus, and then plated at various dilutions on HeLa cell monolayers. Forty eight hours later plaques were counted. Figure 7C shows that at 24 h post-infection approximately twice as many cells from transgenic female mice produced infectious virus particles as from wild-type mice. Real-time PCR analysis [49] further confirmed that at 24 h post-infection the CVB3 genome was present at considerably higher levels in c-FLIP_S_ CD4^+^ and CD8^+^ T cells but not pDC, which do not express the c-FLIP_S_ transgene (Fig.7D). Furthermore we used confocal microscopy to confirm increased presence of CVB3 tagged with GFP in the CD4^+^ and CD8^+^ T cells from infected animals (Fig.S1). We have extended the analysis also to the Jurkat T cell line overexpressing c-FLIP_S_, which demonstrated dramatically increased number of cells with GFP fluorescence (Fig.S1).

### Reduced type I IFN production by CD4^+^, CD8^+^, and pDC cells following CVB3 infection

**Figure 8:**
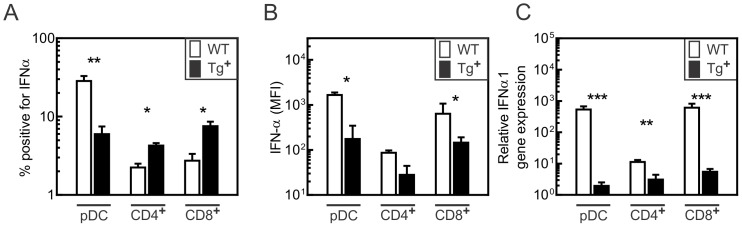
Decreased type I IFN gene expression in cell subsets of c-FLIP_S_ female mice. Flow cytometric analysis of the (A) frequency of IFN-α-positive spleen cells and (B) mean fluorescence intensity (MFI) of IFN-α-positive spleen cells in subsets of pDC, CD4^+^ and CD8^+^ spleen cells obtained from wild-type and c-FLIP_S_ female mice 24 h following CVB3 infection. (C) The relative gene expression of IFN-α1, IFN-α4, and IFN-β was detected with RT-PCR using RNA isolated from the different cell subsets purified by flow cytometric sorting from wild-type and c-FLIP_S_ mice 24 h after CVB3 infection. Data were analyzed using the 2^ΔΔCt^ method using β-actin as the calibrator.

Given that CD4^+^, CD8^+^, and pDC cells become infected with CVB3, we further examined whether these cells also produce type I IFN. Cell subsets were purified from spleens of wild-type and c-FLIP_S_ mice 24 h after infection, and intracellular type I IFN was detected in cells treated with PMA, ionomycin, and Brefeldin A using flow cytometry. The highest type I IFN production following CVB3 infection was observed in wild-type pDC, and nearly 5-fold decreased in pDC from c-FLIP_S_ mice (Fig. 8A). We also observed the production of type IFN in both the CD4^+^ and CD8^+^ cells, which in c-FLIP_S_ mice was significantly decreased in CD8^+^ cells (Fig. 8A). Induction of IFN-α and β gene transcription was determined by RT-qPCR. Since little has been reported regarding type I IFN gene expression in CD4^+^ and CD8^+^ T cells, we tested the expression of IFN-α subtypes as described previously [51]. There was a strong correlation between the levels of mRNA induction and the levels of IFN-α protein observed by intracellular staining (Fig. 8A, B). In c-FLIP_S_ mice the dominant changes for type I IFNs were observed for IFN-α1 gene, which was at least two orders of magnitude lower for both pDC and CD8^+^ cells compared to the equivalent wild-type populations (Fig. 8B).

## Discussion

The current studies show that increased expression of c-FLIP_S_ in T lymphocytes considerably increases CVB3-induced myocarditis and induction of autoimmune, heart-specific CD8^+^ effector T cells, while inhibiting CD4^+^ FoxP3^+^ T regulatory cell responses. We also reveal a profound effect of different c-FLIP isoforms on the induction of type I IFN, likely through differential regulation of caspase-8 activity.

Previous studies have shown a marked sex bias in CVB3 myocarditis susceptibility, with males developing autoimmune CD8^+^ effector T cells and myocarditis when administered 10^2^ or greater PFU of CVB3, whereas females are resistant to disease at these lower virus doses [44], [55], [57], [59]. However, increasing the initial virus inoculum by 100- to 1000-fold in females results in the generation of autoimmune CD8^+^ T cells with substantially increased myocarditis and cardiac viral titers [62]. Thus, myocarditis resistance in females is relative, not absolute, and indicates that females have a substantially higher infection threshold and must internalize considerably more virus than males to induce pathogenicity. Surprisingly, c-FLIP_S_ female mice manifested cardiac viral titers that were increased nearly 1000-fold compared to non-transgenic female mice. This would effectively shift the susceptibility of c-FLIP_S_ female mice into the viral dose range used to confer CVB3-induced myocarditis in male mice [62]. The reason for the enhanced viral load in c-FLIP_S_ female mice likely reflects the severely reduced type I IFN expression in these mice.

The mechanism for promoting autoimmunity in c-FLIP_S_ female mice is likely to be complex. The enhanced autoimmunity in c-FLIP_S_ female mice could not be explained by substantial changes in plasma pro-inflammatory cytokine or chemokine levels subsequent to infection. Analysis of the cytokine/chemokine response in c-FLIP_S_-Tg and control female mice indicates that both groups responded similarly to CVB3 with potent expression of TNF-α and most chemokines. c-FLIP_S_ mice had reduced plasma levels of IL-10 during CVB3 infection, which corresponded to significant decreases in CD4^+^IL-10^+^ cells in the spleen. IL-10 has been shown to suppress autoimmune myocarditis [63], and hence decreasing this cytokine in c-FLIP_S_ mice may promote pathogenesis. Furthermore, IL-10-secreting CD4^+^ cells may represent Tr1 T regulatory cells [64], which can suppress myocarditis induction in CVB3 infected wild-type female mice [65] through inhibition of autoimmune T cell activation [66].

Previous studies have shown that type I IFN responses are protective in CVB3 and other picornavirus infections [5]–[8]. *In vivo* neutralization of type I IFN using anti-IFNα/β greatly enhanced myocarditis and mortality in CVB3-infected mice, whereas treatment of infected mice with exogenous type I IFN abrogated pathology and mortality [5]. Similarly, infection of IFN-α/β receptor-deficient mice resulted in significant increases of virus in serum and liver, leading to enhanced mortality [67]. It has been also shown previously that small interfering RNAs that potentiate type I IFN induction during CVB3 infection, protect mice from development of myocarditis [68].

Type I IFN production during CVB3 infections is triggered mostly by the MDA-5 helicase pathway signaling via MAVS. CVB3 infection of MDA-5^−/−^ or MAVS^−/−^ mice resulted in severely reduced type I IFN response and greatly enhanced organ necrosis and inflammation [8]. We observed that CD4^+^ and CD8^+^ T cells from both wild-type and c-FLIP_S_ mice produced type IFN. The role of type I IFN secretion from T cells is not currently known, although we found that the CD8^+^ subset manifested the same level of type I IFN-α1 gene upregulation as pDC. It was recently shown that type I IFN contributes to the decline of pDCs by mediating their death via the intrinsic apoptosis pathway [69]. This observation is supported by the fact that stimulation of TLR7/9, which also induced type I IFN, also leads to the decline of pDC numbers [70]. The same was observed during certain viral infections such as murine cytomegalovirus (MCMV) [71] and lymphocytic choriomeningitis virus (LCMV) [72]. Decreased numbers of circulating pDCs have been also observed in patients infected with Hepatitis B or C viruses [73], [74] and HIV [75], [76]. We are currently investigating whether the number of pDC is affected by c-FLIP expression and type I IFN during CVB3 infection.

The most striking observation of these studies is that both *in vivo* and *ex vivo* CVB3 infects CD4^+^ and CD8^+^ T cells and the viral load is increased in cells overexpressing c-FLIP_S_. Previously CVB3 RNA was detected in CD4^+^ T cells [77], [78] and Jurkat T cells [79], however no viral RNA was previously observed to our knowledge in CD8^+^ cells. Although our studies did not aim to establish whether the infection of CD4^+^ and CD8^+^ with CVB3 is productive, the fact that the level of VP1 protein following CVB3 infection decreases with time suggests that the infection is transiently productive and can be cleared. Interestingly, the CVB3 RNA is present several days postinfection in the CD4^+^ and CD8^+^ T cells, when the cells do not express any detectable viral proteins. Many studies in mice and humans have demonstrated that although coxsackieviruses are considered highly lytic, persistent CVB3 RNA in host tissues can readily be detected *in vivo* long after infectious virus has been eradicated, during which time viral gene expression is extremely restricted [53], [80]. The presence of persistent or latent CVB3 RNA *in vivo* was shown to have pathological implications on a variety of tissues [81], and even in the absence of virus production, CVB3 RNA can lead to cardiac fibrosis [82]. Studies have indicated that CD8^+^ T cells are a reservoir of circulating HIV-1, resulting in an increase in the activation state of these cells [83]–[85]. Potentially a similar process occurs with CVB3 RNA in CD8^+^ T cells.

The mechanism of how T cells expressing c-FLIPs could affect type-I IFN expression in pDC is currently unknown. It was previously shown that type I IFN, regardless of the source, modulates pDC, and thus fine tunes systemic type I IFN response to viruses [69]. The contribution of pDC to the early innate control of viral infections was shown to be critical against rapidly replicating viruses such as Herpes simplex virus [86], respiratory syncytial virus [87], mouse cytomegalovirus, and vesicular stomatitis virus (VSV) [88]. Conversely, mice depleted of pDC were shown to clear more effectively slower replicating viruses such as LCMV-Armstrong [69]. Interestingly, infection of mice with the Armstrong strain of LCMV was shown to result in inhibition of type I IFN secretion from pDC, even though this LCMV strain infects a minimal proportion of conventional DC and pDC [89]. This suggested that virus replication within pDC is not a prerequisite for their suppression of type I IFN production, but that the exposure of pDC to the infectious environment is likely responsible for this defect [90]. It was also shown that exposure of DC to Herpes simplex virus *in vivo* prevents them from producing IFN-α after re-challenge with the same virus *in vitro* [91].

The observation that c-FLIP_S_ mice present with higher viral load in T cells suggests two explanations. First, it is possible that c-FLIP_S_ protects infected and activated T cells from apoptosis to extend cell survival and therefore preserve the virus in the cells. Second, c-FLIP_S_ could enhance autophagosome formation [92]. It was shown that autophagy in T lymphocytes is indispensable for surviving growth factor withdrawal and regulating the mitochondria and endoplasmic reticulum [93]–[95]. CVB3 is known to employ autophagosomes in its own replication [96], and it was demonstrated that inhibition of autophagosome formation significantly reduced CVB3 replication, and conversely, induction of autophagy by rapamycin or nutrient deprivation resulted in the reverse effect [97]. Of note, a viral-FLIP analogue of c-FLIP_S_ from rhesus monkey rhadinovirus was shown to inhibit apoptosis during active infection by employing autophagosome formation and to regulate viral persistence [98]. Taken together it is plausible that in addition to the ability of c-FLIP_S_ to protect cells from apoptosis and down regulating type IFN, it may also promote autophagosome formation resulting in enhanced CVB3 replication.

It was established that active caspase-8 is required for regulation of the RIG-I helicase pathway downstream of MAVS [27], [29]. c-FLIP_S_ and v-FLIP can heterodimerize with caspase-8 through their mutual death effector domains. However, since c-FLIP_S_ and v-FLIP variants lack the C-terminal activation domain for caspase-8 that is present in c-FLIP_L_, c-FLIP_S_ and v-FLIP block further caspase-8 activation [26], [38], which could serve to turn off type I IFN production via the RIG-I helicase pathway. This model is consistent with our findings. It may also explain why all v-FLIPs isolated to date from a variety of viruses are short FLIPs lacking the C-terminus [26], [33]. Recently, caspase-8-mediated cleavage of RIP1 was demonstrated to negatively regulate the activation of IRF3 in the RIG-I complex, arguing for a central role of caspase-8 and RIP1 not only for cell death but also for inflammatory or antiviral responses [29]. However, as noted in the same study, caspase-8 and RIP1 are also critical for the initial activation of the RIG-I pathway. This suggests a possible complex bimodal effect of these proteins, promoting initial activation of RIG-I followed by its downregulation. That study did not examine the effects of any FLIP isoforms, which might considerably modify the kinetics of this transition. c-FLIP_L_ can alter the substrate preferences of caspase-8, which could include RIP1 [29]. This view is supported by the fact that the substrate specificity of the caspase-8/c-FLIP_L_ complex is an order of magnitude higher for c-FLIP_L_ than for RIP1 [99]. Our findings suggest that the various forms of FLIP confer either different substrate preferences for caspase-8 or possibly different locations within the cell, in association or not with RIP1. In this regard, the greater association of MAVS with c-FLIP_L_ than with c-FLIP_S_ during viral infection may profoundly affect not only the ability of RIG-I, FADD, and RIP1 to translocate to MAVS at the mitochondria, but c-FLIP_L_ may also inhibit cleavage of MAVS by the CVB3 3C^pro^ protease [23]. Our findings thus offer an explanation why it would be preferable for viruses to acquire expression of the short form of FLIP rather than full-length FLIP. Viruses, such as the Kaposi' sarcoma-associated herpes virus (KSHV), can confer low type I IFN secretion, apoptosis resistance, and tumor progression by expression of v-FLIP isoforms that resemble c-FLIP_S_ [100] and are upregulated during late stages of KHSV-induced sarcoma [101].

## Supporting Information

Figure S1 (A) Mice were infected by intraperitoneal injection of 100 PFU of CVB3-GFP. Spleens from wild type and c-FLIP_S_ mice (A) were aseptically harvested 48 h post infection, followed by mechanical homogenization. Erythrocytes were removed by Gey' hemolysis, and the resulting fraction was depleted of B cells by incubation with magnetic anti-CD19 antibodies and separation through a magnetic field. The flow-through was enriched for CD4^+^ and CD8^+^ T cells. These cells were briefly trypsinized and washed with PBS to remove any possible adherent virus, and then applied to poly-lysine-coated glass coverslips. After adherence for 30 min, cells were fixed for 30 min in 2% formaldehyde. After washing, nuclei were counterstained with Hoechst33342, and samples were embedded for microscopy. The cells were analyzed using a Zeiss 510 Meta confocal laser scanning microscope with a 63× objective. (B) The frequency of *in vivo* CVB3-GFP-infected spleen cells was quantified by flow cytometry. Lymphocytes were stained with anti-CD4-PerCp/Cy5.5 and anti-CD8-APC/Cy to identify T cells, or anti-B220-PE/anti-CD11c-APC to identify pDC. Collection gate was set on single lymphatic cells in forward/sideward scatter, and the fluorescence of 20,000 individual events was collected. Cells from mock-infected animals served as negative controls to set the fluorescence threshold for CVB3-GFP detection. The frequency of CVB3-GFP^+^ cells was plotted as percent positive of their respective population. (C) Higher power magnification (63× objective with an additional 4× digital zoom) to show that CVB3-GFP is localized to the cytoplasm. (D) For *in vitro* experiments, human Jurkat cells were stably transfected with a plasmid containing the human c-FLIP_S_ sequence. After selection in puromycin, 5×10^6^ cells were infected with CVB3-GFP (MOI = 10). Samples were prepared 48 h post infection for analysis of GFP-expression as described previously, and analyzed by confocal microscopy.
(TIF)
