# Supplementary figures and images for: c-FLIP-Short Reduces Type I Interferon Production and Increases Viremia with Coxsackievirus B3
Source: PLoS One. 2014 May 9;9(5):e96156. doi: 10.1371/journal.pone.0096156 (PMC4015977; doi:10.1371/journal.pone.0096156)

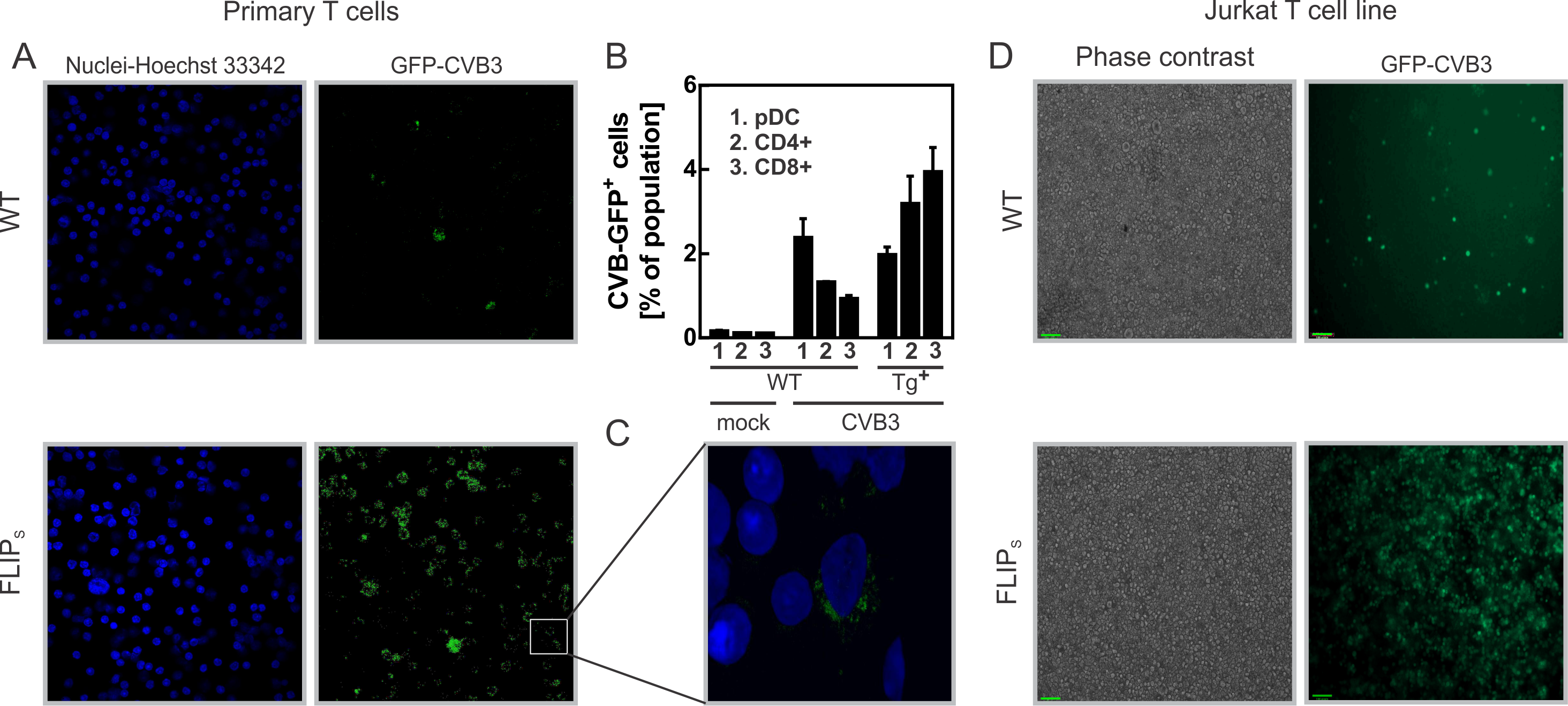

Supplement: Figure S1 — (A) Mice were infected by intraperitoneal injection of 100 PFU of CVB3-GFP. Spleens from wild type and c-FLIPS mice (A) were aseptically harvested 48 h post infection, followed by mechanical homogenization. Erythrocytes were removed by Gey' hemolysis, and the resulting fraction was depleted of B cells by incubation with magnetic anti-CD19 antibodies and separation through a magnetic field. The flow-through was enriched for CD4+ and CD8+ T cells. These cells were briefly trypsinized and washed with PBS to remove any possible adherent virus, and then applied to poly-lysine-coated glass coverslips. After adherence for 30 min, cells were fixed for 30 min in 2% formaldehyde. After washing, nuclei were counterstained with Hoechst33342, and samples were embedded for microscopy. The cells were analyzed using a Zeiss 510 Meta confocal laser scanning microscope with a 63× objective. (B) The frequency of in vivo CVB3-GFP-infected spleen cells was quantified by flow cytometry. Lymphocytes were stained with anti-CD4-PerCp/Cy5.5 and anti-CD8-APC/Cy to identify T cells, or anti-B220-PE/anti-CD11c-APC to identify pDC. Collection gate was set on single lymphatic cells in forward/sideward scatter, and the fluorescence of 20,000 individual events was collected. Cells from mock-infected animals served as negative controls to set the fluorescence threshold for CVB3-GFP detection. The frequency of CVB3-GFP+ cells was plotted as percent positive of their respective population. (C) Higher power magnification (63× objective with an additional 4× digital zoom) to show that CVB3-GFP is localized to the cytoplasm. (D) For in vitro experiments, human Jurkat cells were stably transfected with a plasmid containing the human c-FLIPS sequence. After selection in puromycin, 5×10^6^ cells were infected with CVB3-GFP (MOI = 10). Samples were prepared 48 h post infection for analysis of GFP-expression as described previously, and analyzed by confocal microscopy. (TIF) [file pone.0096156.s001.tif]
